# Intractable pneumothorax due to rupture of subpleural rheumatoid nodules: a case report

**DOI:** 10.1186/s40792-018-0502-8

**Published:** 2018-08-08

**Authors:** Masanori Shimomura, Shunta Ishihara, Masashi Iwasaki

**Affiliations:** Department of General Thoracic Surgery, Ayabe City Hospital, 20-1 Otsuka, Aono-cho, Ayabe, Kyoto 623-0011 Japan

**Keywords:** Secondary pneumothorax, Rheumatoid arthritis, Thoracoscopic surgery, Pleurodesis

## Abstract

**Background:**

In rare cases, rheumatoid pleural nodules can rupture into the pleural cavity to cause pneumothorax or empyema. We report successful surgical treatment of a patient with an intractable secondary pneumothorax due to rupture of a subpleural rheumatoid nodule into the pleural cavity.

**Case presentation:**

A 75-year-old man with a medical history of rheumatoid arthritis, acute coronary syndrome, and diabetes was admitted to our hospital because of left chest pain and dyspnea. A chest X-ray and chest computed tomography (CT) scan showed a left pneumothorax and several small subpleural nodules with cavitation. Repeated pleurodesis via a chest tube failed to improve the pneumothorax, so we decided to perform thoracoscopic surgery. Air leakage was detected in the left upper lobe where the subpleural nodule was visible on chest CT. Resection of the lesion successfully resulted in resolution of the air leakage. The final pathological diagnosis of the subpleural nodule was a pulmonary rheumatoid nodule. The patient has had no evidence of recurrence of pneumothorax after surgery.

**Conclusions:**

We obtained a final pathological diagnosis of a rheumatoid nodule that caused an intractable pneumothorax. Pneumothorax associated with rupture of rheumatoid nodules in the subpleural cavitary is difficult to treat with thoracoscopic surgery as a second-line treatment.

## Background

Rheumatoid arthritis (RA) is the most common form of chronic inflammatory arthritis; RA can cause noncardiac thoracic complications, such as pleuritis, emphysema, pneumothorax, interstitial pneumonia, and rheumatoid nodules [1]. Noncardiac thoracic complications of RA occur in 5–20% of RA patients and account for approximately 20% of RA-associated mortality [2–4]. We report successful surgical treatment of an intractable secondary pneumothorax due to rupture of a subpleural rheumatoid nodule into the pleural cavity.

## Case presentation

**Fig. 1:**
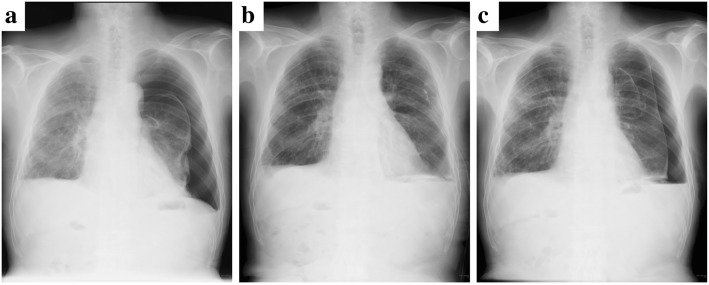
Chest radiograph showing a left pneumothorax on admission (**a**). The lung expansion improved after repeat pleurodesis (**b**). A relapsed left pneumothorax occurred on readmission (**c**)

**Fig. 2:**
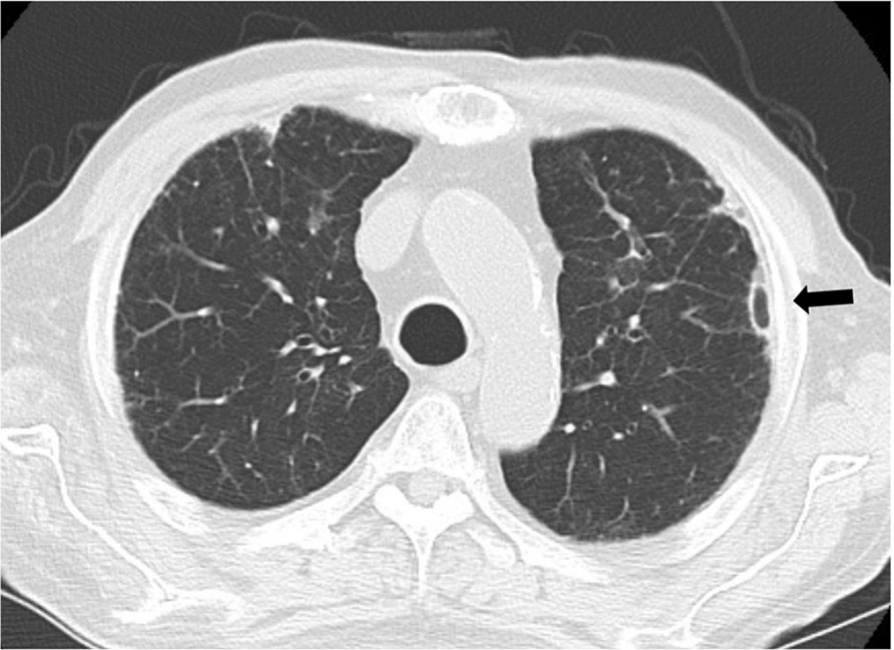
Chest computed tomography before pneumothorax showing a subpleural nodule with cavitation (arrow)

**Fig. 3:**
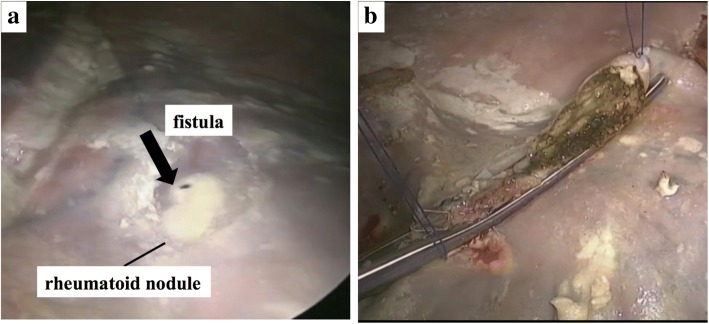
Intraoperative findings of a fistula from the rheumatoid nodule (**a**). The lung parenchyma was sutured continuously after resection of the rheumatoid nodule (**b**)

**Fig. 4:**
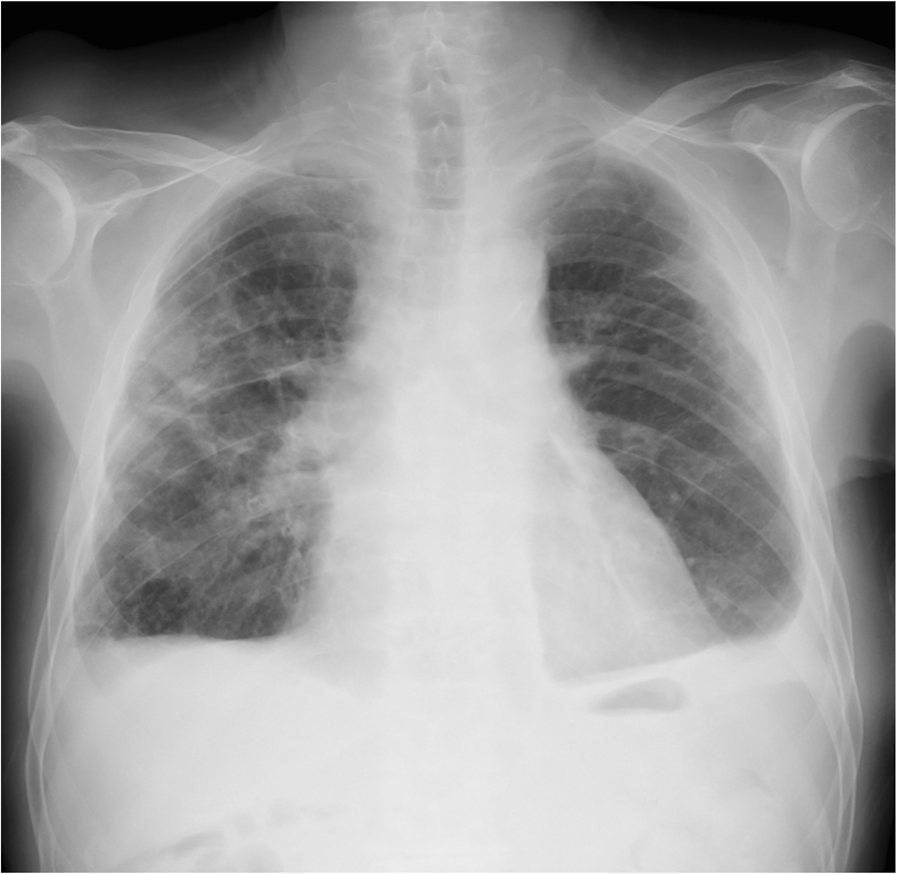
Postoperative chest radiograph showing full expansion of the lung

**Fig. 5:**
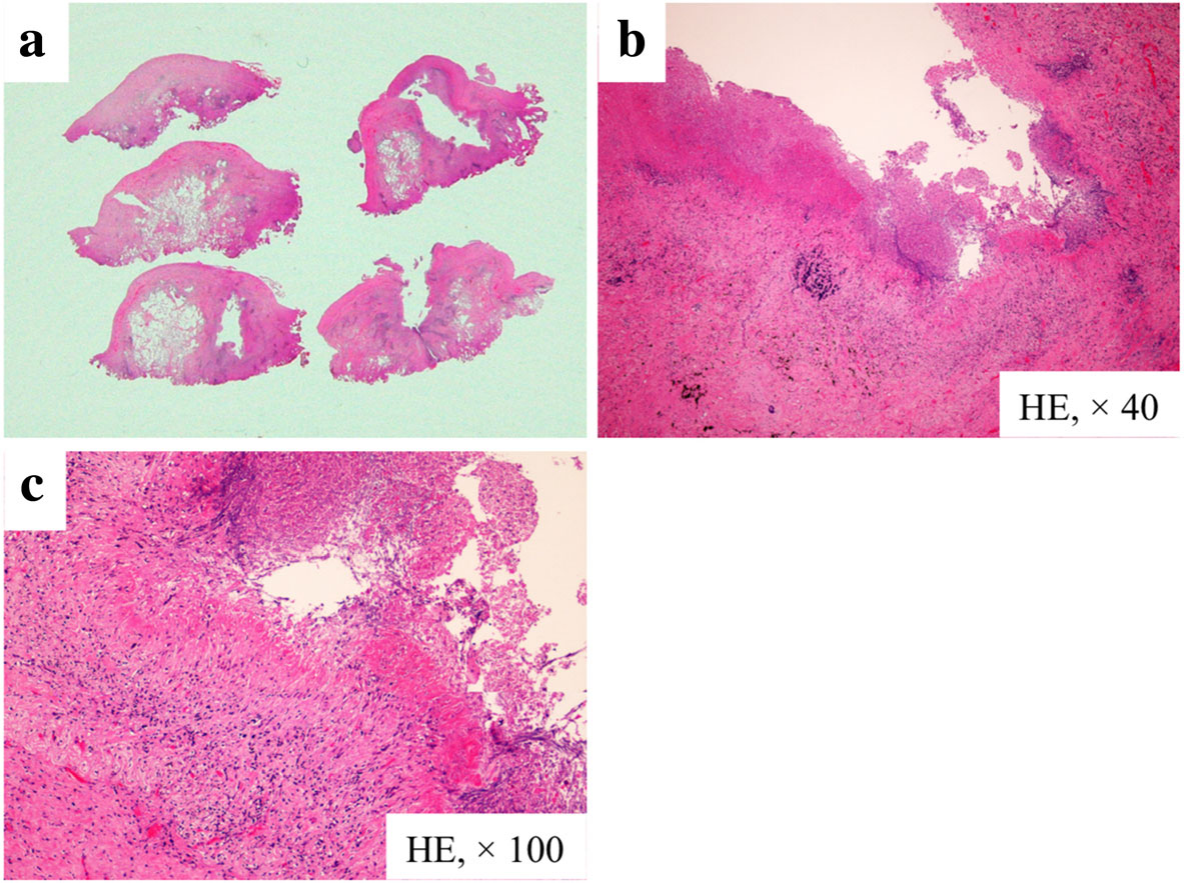
Pathological diagnosis of the resected specimen. A subpleural rheumatoid nodule with cavitation and a defect in the pleura where the cavity had formed (**a** loupe image). A highly magnified view of the lesion showing fibrinoid necrosis surrounded by histiocytes and lymphocytes (**b** HE, × 40; **c** HE, × 100)

A 75-year-old man was admitted to our hospital because of left chest pain and dyspnea. A chest X-ray showed a left pneumothorax (Fig. 1), and a previous chest computed tomography (CT) scan revealed a subpleural pulmonary nodule with cavitation in the left upper lobe (Fig. 2). The patient had been on prednisolone and methotrexate for rheumatoid arthritis. He also had a past history of acute coronary syndrome 5 years previously; this was treated with coronary stent implantation followed by anticoagulant therapy. He additionally had a past history of a right pneumothorax 3 years previously that was presumptively caused by rupture of a subpleural rheumatoid nodule and successfully treated with conservative thoracic drainage. Regarding his present admission, the left pneumothorax did not resolve after 1 week of chest tube drainage. There were no bacterial pathogens present in the pleural effusion. We decided to perform pleurodesis because his left lung expanded well after chest drainage and he had some risks for pneumothorax surgery, as previously mentioned. The air leakage finally subsided after six times pleurodesis procedures (with a blood patch once, a talc slurry three times, and OK-432 (streptococcal preparation) two times) prior to discharge (Fig. 1b). However, he presented to our hospital 1 week after being discharged because the pneumothorax had asymptomatically relapsed (Fig. 1c). Thus, we decided to perform thoracoscopic surgery to resect the subpleural lesion that caused intractable pneumothorax 2 months after the first admission. The surgery was performed through the fourth intercostal space along the midaxillary line, sixth intercostal space along the anteroaxillary line, and seventh intercostal space along the posteroaxillary line. There were rigid adhesions on the apex, mediastinum, and diaphragm due to repetitive pleurodesis. A sealing test revealed a pleural fistula with air leakage of the left upper lobe where the previous chest CT scan had demonstrated a small subpleural nodule with cavitation (Fig. 3a). The visceral pleura was so thick and hard that we had to resect the lesion with surgical scissors instead of with automatic staplers. The resected lung parenchyma was continuously sutured with 4-0 Prolene® (Ethicon endo-surgery) and covered with a polyglycolic acid sheet (Fig. 3b). There was no air leakage at the end of surgery. The lung gradually expanded (Fig. 4) for a few days. The chest tube was successfully removed on the third postoperative day, and the patient was discharged on the seventh postoperative day. The final pathological diagnosis was a pulmonary rheumatoid nodule on the pleura (Fig. 5). The patient has had no evidence of recurrence of pneumothorax during 1 year of follow-up.

### Discussion

Pulmonary rheumatoid nodules occur in less than 1% of patients with RA [5]. Rheumatoid nodules can rupture into the pleural cavity and cause pneumothorax, empyema, and bronchopleural fistula (BPF). Some reports also indicate that rapid growth of rheumatoid nodules may be associated with azathioprine treatment [6] or biological therapies [7] of RA. Kim et al. also reported a case of recurrent pneumothorax after etanercept therapy induced by the progression of interstitial lung disease [8]. Pneumothorax caused by noncardiac intrathoracic complications of RA can be resistant to standard care because RA patients are immunosuppressed, which predisposes them to infection and poor healing. Some authors have reported difficulties in performing surgical intervention for pneumothorax induced by RA. Rueth et al. reported that some patients with RA-induced pneumothorax and BPF required long-term care and had pneumothoraces that were resistant to repeat talc pleurodesis [9]. The success rate for pleurodesis via thoracoscopic surgery for the management of pneumothorax in patients without RA is 97%, and the long-term recurrence rates range from 5 to 9% [10]. Regarding pneumothorax in patients with RA, we hypothesize that the control rate for pneumothorax is lower than in those without RA because RA patients have poor wound healing and chronic local inflammation in the causative lesions. Due to the intrathoracic rigid adhesions that form surrounding the causative fistula after repetitive pleurodesis, pleurodesis can be useful for treating patients with multiple rheumatoid nodules because this procedure can prevent recurrent pneumothorax resulting from subsequent rupture of other nodules. On the other hand, surgery to treat any residual air leakage is very difficult because of adhesions that have formed at other locations not contributing to the air leakage and due to the thick visceral pleura. A wide pleural covering technique with an oxidized regenerated cellulose mesh [11] or a polyglycolic acid sheet can be used to treat intractable pneumothorax rather than fistula resection and suturing in patients in whom the cause of pneumothorax are still unknown at the time of surgery. The use of a latissimus dorsi muscle flap placed into the pleural cavity can also be effective because this flap is made with a large muscle with a reliable blood supply and is relatively easy to harvest. Bronchial occlusion with a spigot is also one of the most effective techniques for patients with poor conditions for surgery [12]. In our patient, the causative lesion was suspected to be solitary, and it was difficult to treat the air leakage with bronchial occlusion because the lesion was located in the peripheral lung parenchyma. Additionally, we selected surgical resection to pathologically demonstrate that the definitive etiology of the pneumothorax was rupture of a rheumatoid nodule. In RA patients, it is important to obtain a pathological diagnosis of the underlying cause of pneumothorax because these patients have higher risks of lung malignancies than the general population [13].

## Conclusions

We obtained a final pathological diagnosis of a rheumatoid nodule that caused an intractable pneumothorax. Pneumothorax associated with rupture of rheumatoid nodules in the subpleural cavitary is difficult to treat with thoracoscopic surgery as a second-line treatment.

## Abbreviations

BPF: Bronchopleural fistula
CT: Computed tomography
RA: Rheumatoid arthritis

## References

[1] Chansakul T, Dellaripa PF, Doyle TJ, Madan R (2015). Intra-thoracic rheumatoid arthritis: imaging spectrum of typical findings and treatment related complications. Eur J Radiol.

[2] Lynch DA (2009). Lung disease related to collagen vascular disease. J Thorac Imaging.

[3] Tansey D, Wells AU, Colby TV, Ip S, Nikolakoupolou A, du Bois RM (2004). Variations in histological patterns of interstitial pneumonia between connective tissue disorders and their relationship to prognosis. Histopathology.

[4] Minaur NJ, Jacoby RK, Cosh JA, Taylor G, Rasker JJ (2004). Outcome after 40 years with rheumatoid arthritis: a prospective study of function, disease activity, and mortality. J Rheumatol Suppl.

[5] Hull S, Mathews JA (1982). Pulmonary necrobiotic nodules as a presenting feature of rheumatoid arthritis. Ann Rheum Dis.

[6] Kellet CV, Navarrete RA, Bombardieri SG, Manriquez J (2015). Azathioprine-induced accelerated cutaneous and pulmonary nodulosis in a patient with rheumatoid arthritis. An Bras Dermatol.

[7] Kovacs A, Baksay B, Cserenyecz A, Molnar K, Takacs M, Szekanecz Z (2015). Occurrence of pulmonary rheumatoid nodules following biological therapies. Clin Rheumatol.

[8] Kim SH, Choi SJ, Seo YH, Kim JH, Jeong IW, Sohn SB (2014). Recurrent pneumothorax after etanercept therapy in a rheumatoid arthritis patient: a case report. Chonnam Med J.

[9] Rueth N, Andrade R, Groth S, D'Cunha J, Maddaus M (2009). Pleuropulmonary complications of rheumatoid arthritis: a thoracic surgeon’s challenge. Ann Thorac Surg.

[10] Doddoli C, Barlesi F, Fraticelli A, Thomas P, Astoul P, Giudicelli R (2004). Video-assisted thoracoscopic management of recurrent primary spontaneous pneumothorax after prior talc pleurodesis: a feasible, safe and efficient treatment option. Eur J Cardiothorac Surg.

[11] Kurihara M, Kataoka H, Ishikawa A, Endo R (2010). Latest treatments for spontaneous pneumothorax. Gen Thorac Cardiovasc Surg.

[12] Ueda Y, Huang CL, Itotani R, Fukui M (2015). Endobronchial Watanabe spigot placement for a secondary pneumothorax. J Bronchology Interv Pulmonol.

[13] Simon TA, Thompson A, Gandhi KK, Hochberg MC, Suissa S (2015). Incidence of malignancy in adult patients with rheumatoid arthritis: a meta-analysis. Arthritis Res Ther.

